# Predicting Cardiopulmonary Arrest with Digital Biomarkers: A Systematic Review

**DOI:** 10.3390/jcm12237430

**Published:** 2023-11-30

**Authors:** Gioacchino D. De Sario Velasquez, Antonio J. Forte, Christopher J. McLeod, Charles J. Bruce, Laura M. Pacheco-Spann, Karla C. Maita, Francisco R. Avila, Ricardo A. Torres-Guzman, John P. Garcia, Sahar Borna, Christopher L. Felton, Rickey E. Carter, Clifton R. Haider

**Affiliations:** 1 Division of Plastic Surgery, Mayo Clinic, Jacksonville, FL 32224, USA; ajvforte@yahoo.com.br (A.J.F.);; 2 Department of Cardiovascular Medicine, Mayo Clinic, Jacksonville, FL 32224, USA; 3 Department of Health Sciences Research, Mayo Clinic, Jacksonville, FL 32224, USA; 4 Department of Physiology and Biomedical Engineering, Mayo Clinic, Rochester, MN 55902, USA

**Keywords:** physiological marker, vital signs, electrocardiogram, physiological monitoring, telemetry, cardiac arrest, in-hospital cardiac arrest

## Abstract

(1) Background: Telemetry units allow the continuous monitoring of vital signs and ECG of patients. Such physiological indicators work as the digital signatures and biomarkers of disease that can aid in detecting abnormalities that appear before cardiac arrests (CAs). This review aims to identify the vital sign abnormalities measured by telemetry systems that most accurately predict CAs. (2) Methods: We conducted a systematic review using PubMed, Embase, Web of Science, and MEDLINE to search studies evaluating telemetry-detected vital signs that preceded in-hospital CAs (IHCAs). (3) Results and Discussion: Out of 45 studies, 9 met the eligibility criteria. Seven studies were case series, and 2 were case controls. Four studies evaluated ECG parameters, and 5 evaluated other physiological indicators such as blood pressure, heart rate, respiratory rate, oxygen saturation, and temperature. Vital sign changes were highly frequent among participants and reached statistical significance compared to control subjects. There was no single vital sign change pattern found in all patients. ECG alarm thresholds may be adjustable to reduce alarm fatigue. Our review was limited by the significant dissimilarities of the studies on methodology and objectives. (4) Conclusions: Evidence confirms that changes in vital signs have the potential for predicting IHCAs. There is no consensus on how to best analyze these digital biomarkers. More rigorous and larger-scale prospective studies are needed to determine the predictive value of telemetry-detected vital signs for IHCAs.

## 1. Introduction

In the United States, there are approximately 290,000 adult IHCAs annually, with reported survival rates after hospital discharge between 17% and 18% [1,2,3], with lower than a one-year survival [4].

Similar to metabolic and genetic biomarkers, digital biomarkers are indicators that may be utilized to detect pathogenic processes, normal biological processes, or intervention responses [5,6]. Vital signs are the most crucial physiological indicators of health, and by using telemetry, they turn into digital signatures and biomarkers of disease.

Vital signs represent the easiest, cheapest, and likely the most significant data on hospitalized patients, especially in the acutely ill [7]. Furthermore, evidence demonstrates that abnormal vital signs commonly appear a few hours before IHCAs and that the number and severity of abnormal vital signs before CAs are directly proportional to mortality [8,9]. Consequently, many IHCAs are believed to be preventable [10], and the early recognition of the causes of CA by the healthcare team has been demonstrated to improve survival [11].

In the hospital setting, vital signs serve as the primary foundation for clinical decision making. Precise and timely access to vital signs data is essential to make prudent therapeutic choices. The collection and documentation of this clinical information, which is crucial for patient care, consume a considerable portion of the workday for healthcare professionals [12].

Telemetry is a valuable tool that helps medical staff provide early warning signals. As a result, telemetry is the standard of care and has been increasingly implemented in various units inside medical facilities outside the conventional critical care setting since its introduction more than 50 years ago [13]. Moreover, for individuals experiencing CAs, telemetry use is a crucial element linked to enhanced survival [14]. However, despite the overall positive impact that telemetry appears to have on patient safety, study findings point to unforeseen consequences of excessive monitoring. For instance, alarm fatigue is a well-researched and verified effect [15,16].

Several published criteria with variable vital sign combinations are used to identify early adverse outcomes, including CA [17]. The most well-known are the Early Warning Scores Systems (EWSs), which have been gradually employed in the acute medical setting after showing a predictive potential for CA within 48 h, with an area under the receiver operating characteristic curve of 0.74–0.86 [18]. Yet, despite their remarkable predictive value, EWSs have been limited in some instances by the imprecision and intermittent nature of nurse recording [19,20]. For example, the methods used by doctors to assess respiratory rate have been described to be inaccurate, potentially resulting in a negative impact on patient care and subsequent outcomes [21]. Similarly, the vital signs-based monitoring of central shock is known to lack sensitivity or specificity [22]. Thereby, the potential benefits of the telemetry systems, which offer accurate real-time monitoring for the primary indicators used in healthcare, are demonstrated [23].

With the advent of AI/ML, the healthcare system has been continuously aiming to adopt new tools to improve outcomes in the medical setting. Executing large-scale patient data analysis and deriving predictive models with ML algorithms have demonstrated increasing accuracy in predicting clinical deterioration, with the potential to decrease false alarms and improve patient outcomes [24,25,26]

To our knowledge, this is the first systematic review of the utility of digital biomarkers in predicting IHCAs.

This systematic review aims to answer the following questions:

1. What are the vital sign events or patterns assessed by telemetry that precede IHCAs?
2. What single-vital signs patterns detected by telemetry most accurately predict IHCAs?

## 2. Methods

### 2.1. Eligibility Criteria

#### 2.1.1. Population and Interventions

We included studies conducted on hospitalized patients who suffered IHCAs, excluding studies performed on subjects with out-of-hospital CAs or a history of cardiac transplant. In addition, we restricted our inclusion to only adult patients (≥18 years old). Studies with IHCAs as an outcome were eligible for inclusion if they used bedside telemetry systems for the monitorization, for a minimum of one hour, of at least one of the digital biomarkers of interest (heart rate, blood pressure, respiratory rate, temperature, oxygen saturation, and ECG metrics), reporting data about frequencies, means, or other types of descriptive methods or performance in relation to CAs. Additionally, we excluded studies if they had fewer than 20 subjects or if they used vital sign data starting after the first CA.

#### 2.1.2. Study Types

Studies with the following designs were considered for inclusion: interventional and observational studies, including case series, case controls, and prospective studies. We excluded editorials, reviews, and commentaries.

### 2.2. Information Sources and Search Strategy

We utilized four electronic databases to run our search: PubMed, EMBASE/MEDLINE, Google Scholar, and Web of Science. We used the Preferred Reporting Items for Systematic Reviews and Meta-Analyses (PRISMA in Supplementary Materials) 2020 statement as the basis of our organization [27]. We utilized Boolean expressions to create a complex search strategy. In Supplementary File S1, the search strategy is detailed along with the date when the search was performed. It must be highlighted that only the first 10 pages from the Google Scholar search were screened. The date and language of publication did not limit the search.

### 2.3. Selection and Data Collection Process

The first two authors performed the search independently. The duplicates were then identified and removed using EndNote v20 (Clarivate Analytics). After screening the papers based on titles and abstracts, we retrieved the full text of the relevant articles, which were assessed by full-text readings according to the eligibility criteria. Disagreements among the first two authors were addressed by the decision of the third or fourth authors.

For each study, the following information was obtained: authors, year of publication, design, study goals, population and setting, eligibility criteria, sample size, duration of studies, interventions, demographics, telemetry systems used, vital signs assessed, total monitoring hours, frequency of manual or automated measurements, other clinical characteristics (e.g., hospital units, baseline characteristics, relevant medical history), vital signs data (e.g., absolute values, thresholds, cut-off values, trends, slopes, and other described characteristics), any CA prediction or association effect measures, limitations, funding, and conflict of interest information.

### 2.4. Risk of Bias of Individual Studies

The first two authors independently evaluated each study for bias using the ROBINS-I tool from the Cochrane Library for nonrandomized studies [28]. Any conflicts among the first two authors were solved by the decision of the third or fourth authors independently. Thereafter, a summary and a graph were created using RevMan 5.4 (Cochrane Collaboration), enabling the stratification of bias in diverse areas.

### 2.5. Synthesis Methods

After a comprehensive review of the included studies, the most significant data from the methodologies, variables, outcomes, and performance were tabulated using the Microsoft Excel spreadsheet software version 2016 to display the results of each study. Finally, the narrative synthesis was performed after tabulating the results, describing the differences between studies regarding their methodologies, interventions, and aims.

## 3. Results

### 3.1. Study Selection and Characteristics

**Figure 1:**
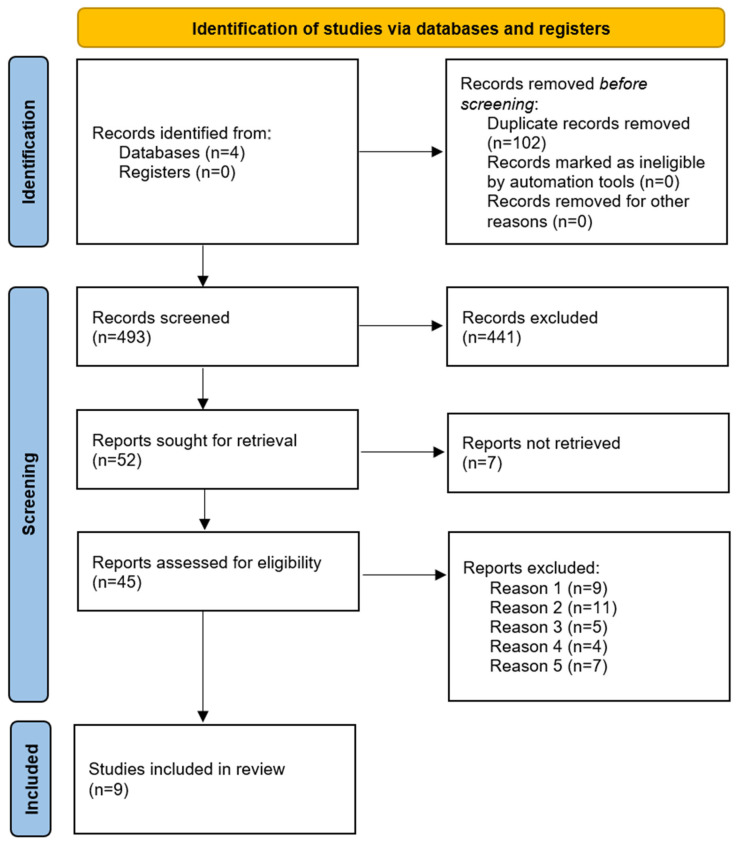
Study selection process: Process of study selection following the PRISMA 2020 flow diagram for systematic reviews.

The search provided 595 studies, yielding 493 after the removal of duplicates. After that, 441 were excluded after initial screening by titles and abstracts. The resulting 45 full-text articles were retrieved and assessed, making 9 articles eligible for inclusion (Figure 1). From the excluded studies, 9 did not mention the use of telemetry; 11 did not assess CA as a single outcome; 5 did not report information about vital signs/ECG; 4 met the exclusion criteria (2 with <20 subjects and 2 with <18-year-old patients); and 7 were found to be irrelevant.

We found 9 retrospective observational studies eligible for inclusion (Table 1), subcategorized into 7 case-control studies [29,30,31,32,33,34,35] and 2 case series [36,37]. From these studies, 5 described vital signs, including respiratory rate, blood pressure, temperature, and oxygen saturation (Table 2) [29,30,33,34,35]. The remaining 4 studies described ECG parameters (Table 3) [31,32,36,37]. Only 2 studies included patients from ≥2 healthcare centers [31,36].

There was a remarkable heterogeneity between the studies in methodology, population, time of data collected before CA, outcomes, and endpoints. All the studies collected the data for analysis from electronic health records and described their findings differently. The types of CA studied and the definition of CA varied among studies. In general, the studies differed in the way they assessed the ECG. Seven studies assessed their parameters of interest until the time of CA [31,32,33,34,35,36,37]; instead, Churpek et al. (2012) [29,30], in both studies, excluded the last 30 min of data since their objective was to predict the event in a time to intervene. Two studies were conducted using only data from the ICU [34,35]. Some studies matched their control subjects [30,31,32,35], although the control matching criteria differed. In patients who suffered a CA more than one time during their hospitalization, all the studies consistently collected only the data before the first arrest.

### 3.2. Results for Individual Studies

The specific results for each study are detailed and organized in Table 1, Table 2 and Table 3.

From those assessing ECG parameters, Do et al. (2015) compiled data from three ECG leads every hour and then at 90, 50, 40, 30, 20, 10, 5, 2, and 1 min before CA, to analyze changes from baseline of five metrics and describe their frequency [37]. Instead, Attin et al. (2015) conducted their study including only patients with a history of structural heart diseases and analyzed five metrics using data from seven ECG leads, with 10 s of disclosures of ECG each hour before CA, with an additional 15 min of screening before CA looking for heart rate changes [36]. Hu et al. (2013) [32] reported results based on one lead, analyzing both upper and lower thresholds of eight metrics that would activate alarms in their patient set, achieving the highest true positive rate (TPR) with the lowest false positive rate (FPR) possible. Given the large number of variables reported by the authors, we only reported the results of the absolute slope of the trending at the window showing the highest TPR, excluding those with TPR = 0% [32]. Similarly, Ding et al. (2015) [31] used an ECG analysis program to generate 17 metrics automatically to evaluate the longest trend duration, upper and lower thresholds of terminal value, and slope. Next, they tested the alarm activation in their patient set under the established maximum FPR. We only describe the results at a maximum FPR of 5% with a TPR greater than 10%. Additionally, in the “other parameters” column, we reported the six variables with the highest TPR, which were trend duration parameters [31].

From those which focused on digital biomarkers other than ECG, the two studies conducted by Churpek et al. (2012) [29,30] used the same population in the case group from the ICU and other telemetry units but with a different number of controls [29,30]. In the first one, they collected the maximum and minimum values achieved on each vital sign, determined cut-off values, and calculated the performance predicting CA. Additionally, they calculated the change from baseline [29]. In the second study, they derived, internally validated, and tested a CA prediction model using only the three parameters that achieved the highest performance, reporting the frequency of each maximum and minimum value of the vital signs assessed within the subjects [30].

On the other hand, Rozen et al. (2014) [35] assessed vital signs from ICU patients with no limitation of medical therapy in place, choosing a different cut-off for each significant and reporting the median and interquartile range for the highest and lowest values achieved [35]. Oh et al. (2016) [34] calculated the mean values of hourly vital signs of patients who died of CA in the ICU. Finally, McGrath et al. (2021) [33] analyzed the mean and range of percentage differences of two variables between cases and controls using 5-min and 1-h intervals. We reported the results only at the 0–5 and 21–25 intervals. Additionally, they analyzed percentage differences within the case group using a baseline hour to compare to the following hours; we only showed the 1st h vs. 7th, which showed the highest differences.

## 4. Discussion

In this systematic review, we identified nine eligible retrospective studies that used telemetry to assess vital signs patterns preceding IHCA.

**Figure 2:**
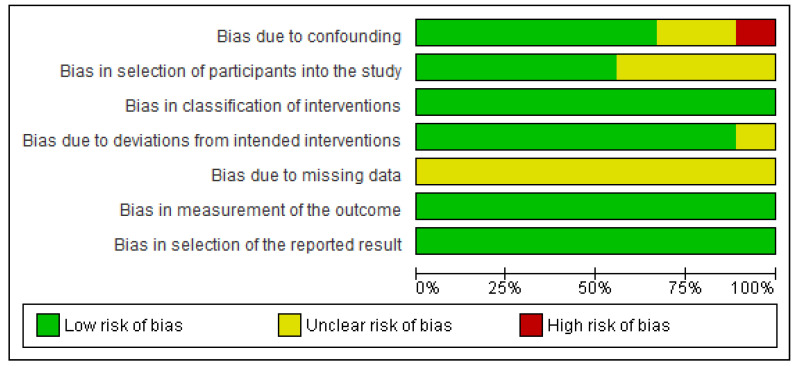
Risk of bias graph: Review authors’ judgments about each risk of bias item presented as percentages across all included studies.

**Figure 3:**
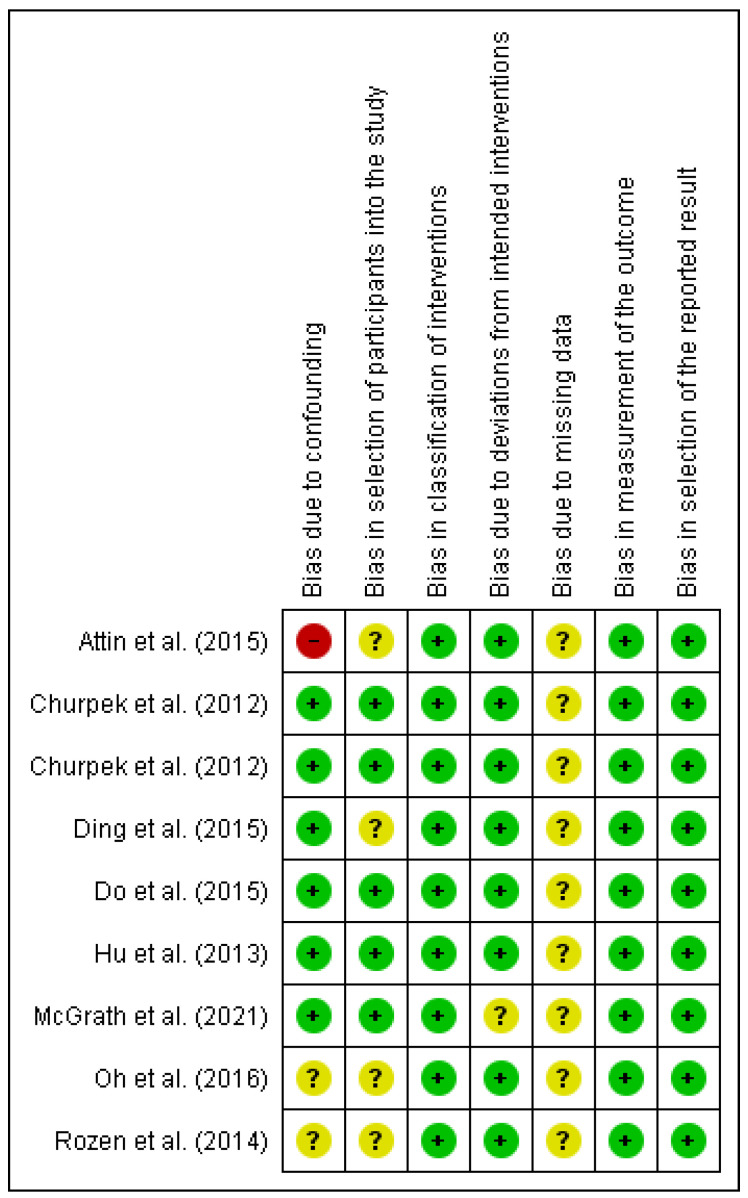
Risk of bias summary: Review authors’ judgments about each included article’s risk of bias in the different components of the analysis. Red stands for high risk of bias, yellow stands for unclear risk, and green stands for low risk [29,30,31,32,33,34,35,36,37].

**Figure 4:**
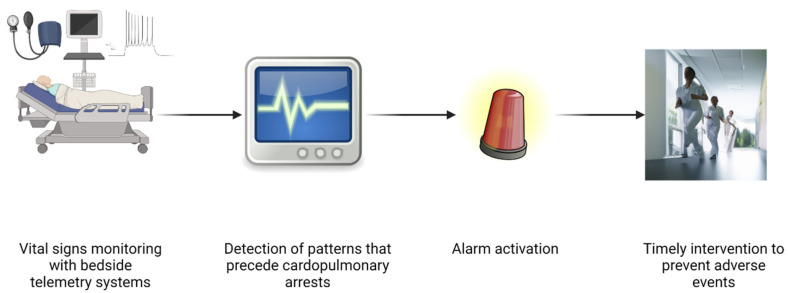
Monitoring digital biomarkers to predict cardiac arrest. This image depicts the use of telemetry to monitor and capture vital sign changes preceding cardiac arrests. Alarms trigger rapid response systems to intervene in a timely manner. Created with BioRender.com.

Our main findings highlight the lack of solid evidence on which vital signs most accurately predict IHCA; some limited evidence suggests that some vital signs can be more predictive than others. However, the risk of bias within and across studies was deemed moderate (Figure 2 and Figure 3), for which more well-designed multicentric studies are still required to increase the validity of the results. The results from the included studies suggest that changes detected by telemetry can occur within a timespan sufficient for timely intervention, as shown in Hu et al. (2013) [32], where the earliest change was detected approximately 20 h before the CA. We suggest that increased research in this area could result in more precise and valid findings that could facilitate the standardization of pre-arrest patterns to make telemetry systems capable of predicting cardiac arrests with high accuracy and enough lead time for timely intervention by rapid response teams. Figure 4 depicts the ideal scenario where telemetry systems aid healthcare staff in improving the quality of care and outcomes.

We further discuss the findings and implications of each digital biomarker in the section below.

### 4.1. Heart Rate

Heart rate was the vital sign most assessed in the studies. However, it is difficult to determine the heart rate pattern before CA since each study used a different methodology. Bradycardic events are known to occur 10 min before an imminent IHCA [38]. Accordingly, Attin et al. (2015) [36] determined that 82% of patients had a low heart rate before CA; yet, they specifically studied patients with structural heart diseases, which might not represent the changes observed in the general hospitalized population. In contrast, Churpek et al. (2012) [29,30] indicated that the maximal heart rate was a significant predictor of CA, whereas minimum heart rate did not achieve statistical significance; however, they collected the data until 30 min before CA, excluding a critical timeframe for the occurrence of bradycardia. Moreover, McGrath et al. (2021) [33] and Oh et al. (2016) [34] found that the heart rate was higher before CA vs. controls, although only Oh et al. (2016) [34] found significance when comparing CA patients vs. non-CA patients who were later discharged.

### 4.2. Blood Pressure

Systolic blood pressure has been previously stated as a common change observed over the hours leading up to the arrest [39,40], and it is part of the traditional Modified Early Warning Score (MEWS) and National Early Warning Score (NEWS) [41,42]. Oh et al. (2016) [34] and Rozen et al. (2014) [35] reported differences in systolic blood pressure among groups; in fact, systolic blood pressure was the only digital biomarker in Oh et al.’s study that showed statistical differences compared to both control groups [34]. However, such differences were probably not clinically significant compared to the non-CA patients who died in the ICU. In contrast, Churpek et al. (2012) [29,30] determined that only the diastolic blood pressure was a significant predictor of CA, hence its inclusion in the CART (Cardiac Arrest Risk Triage) score, which has shown higher predictive potential than the widely used MEWS [43]. Additionally, Rozen et al. (2014) [35] determined that mean arterial pressure also showed significant differences among groups, suggesting its predictive potential for CAs.

### 4.3. Respiratory Rate

Numerous studies have shown that an elevated respiratory rate is a common precedent of impending CA [40,44,45]. Accordingly, the four studies evaluating respiratory rate showed its relevance as a predictive variable. From them, three studies described the statistical significance of findings compared to controls; the remaining did not report it. Churpek et al. (2012) [29] and Rozen et al. (2014) [35] found differences in maximal respiratory rates among the groups, whereas Oh et al. (2016) [34] found mean respiratory rate differences between cases and discharged patients. Furthermore, Churpek et al. (2012) [29] explained that respiratory rate (especially maximal respiratory rate) behaved as the best vital sign predictor of IHCA. Other authors suggest that respiratory rate is the best predictor of CA after finding that CA patients were 5.56 times more likely to have a respiratory rate greater than 27 bpm in the previous 72 h (95% CL = 2.67–11.49) [45].

### 4.4. Oximetry

The oxygen saturation is a variable assessed in the NEWS, which has been deemed to predict IHCA and mortality among those who suffer an IHCA [46,47]. Churpek et al. (2012) [29] and Rozen et al. (2014) [35] did not find significant differences between cases and controls. However, McGrath et al. (2021) [33] determined its usefulness in predicting imminent IHCA. Still, due to the type of IHCA included in their study, we suggest that oxygen saturation might be a potential predictor of pulseless electrical activity (PEA) [33].

### 4.5. Temperature

The underlying cause of CA may sometimes be identifiable and reversible, such as severe hypothermia [48]; however, it usually occurs in patients with body temperatures below 30 °C, and the CA usually takes place out of the hospital. Churpek et al. (2012) [29] and Oh et al. (2016) [34] determined that body temperature is a poor predictor of IHCA. Moreover, body temperature has been defined as a weak predictor of CA that should not be included in prediction models [49].

### 4.6. ECG

Although ECG metrics are not easily recognizable in monitors of telemetry systems, there are a wide variety of parameters that can be assessed for their predictive potential for IHCA. We showed different studies accounting for more than 20 parameters; however, no metrics achieved high sensitivities at the chosen cut-off values since the goal was to keep an FPR close to 0% to maintain a low false alarm rate [31,32]. However, Do et al. (2015) [37] reported that 77% of the participants had an ECG abnormality during the 24 h preceding the IHCA. Furthermore, despite other authors not having found enough prediction accuracy of ECG [50], its capability has been further confirmed by using deep learning algorithms, achieving AUC > 0.90 for the prediction of IHCAs within 24 h [51].

### 4.7. Implication of Results

This systematic review suggests that there is not a single or combination of ECG metrics or vital sign values found in all patients, as each patient differs in baseline clinical status, undergoing medications, and causes of CA. It is crucial to acknowledge the nuanced complexities introduced by comorbid conditions, in which altered physiological parameters may affect the reliability and accuracy of digital biomarker data. It is imperative that clinicians assess the implications of digital biomarkers on a case-by-case basis, considering the individual patient’s comorbid conditions and their potential influence on data interpretation. Despite these challenges, we were able to show the high prevalence of such physiological abnormalities detected by telemetry preceding IHCAs. Moreover, some studies identified the most reliable predictors of CA to derive predictive models that provided greater accuracy than single digital biomarkers [29,30,31,35].

This review also suggests that although a single vital sign can achieve a high specificity to predict CA at the expense of low sensitivity, a combination of the most accurate, sometimes along with other clinical or demographic data, can achieve a significant accuracy. We propose that the combination of the most accurate vital signs may offer the potential to modulate alarms to decrease the before-mentioned alarm fatigue issue associated with telemetry.

Telemetry devices with alarm systems are regularly used in hospital care units to continuously monitor and display patients’ vital signs. Alarm fatigue can occur when too many alarms are generated, resulting in nurses and physicians disabling or disregarding them and potentially leading to negative consequences [52,53,54]. Still, healthcare benefits from telemetry by increasing the accuracy of vital signs measurements, especially those hardly detected by current telemetry systems [55].

### 4.8. Early Warning Scores

Early warning scores have been widely studied and used in the USA to predict cardiac arrest achieving varied levels of accuracy [18,56]. The parameters as well as the cut-off values included in the EWS systems were developed decades ago by expert consensus and modified and improved upon a test-fail basis until an acceptable system, able to serve as a track a trigger system to identify early signs of deterioration was developed. However, there have been multiple revisions to these EWS systems due to inconsistencies in their performance across different settings [57].

A more in-depth understanding of the factors that precede CA could play a significant role in enhancing the accuracy of these predictions. For instance, two of the most widely used EWS systems, MEWS and NEWS, utilize temperature as one of the parameters for their composite scoring. Contrarily, our review indicates that temperature is a poor predictor of CA [29,34,49]

Historically, EWS systems were primarily based on clinical data and static vital sign values. This approach was largely influenced by the constraints of the pre-digital era, where real-time data analytics and dynamic monitoring were not readily available. However, with the advent of modern technological advancements and the widespread adoption of electronic health records, there is an opportunity to integrate more sophisticated and predictive metrics into these systems. For instance, vital sign trends, which have previously demonstrated superiority in predicting cardiac arrest when compared to static values [58]. In this study, we have identified some metrics that hold promise. Specifically, the slope of the ECG segment and the trend duration emerged as particularly significant indicators [31,32].

### 4.9. Telemetry and AI/ML

As we progress into the future of cardiac care, we anticipate a marked improvement in the prediction of IHCAs. This improvement will likely be driven by refined predictive models augmented with AI/ML tools [59]. Several studies have underscored the potential of AI/ML in predicting cardiac arrest, consistently outperforming EWS systems [60,61,62].

One of the primary concerns with AI, despite its demonstrated efficacy, is the “black box” nature of its results. AI models are inherently complex, and in healthcare settings, understanding the underlying rationale behind the decisions is crucial [63]. This can lead to a lack of trust by clinicians, particularly in situations like predicting cardiac arrest, a life-critical and time-sensitive scenario [64].

By delving deeper into the vital signs that usually precede a cardiac arrest, clinicians can bolster their confidence in the outputs generated by AI models. This notion further emphasizes the significance of accurately identifying and evaluating the variables that should be incorporated into these models.

The volume of healthcare data currently available for model training is immense. As the dimensionality of this data escalates, so does the computational expense of training models, often growing exponentially. Addressing this complexity demands strategies to reduce the dimensionality on the data under analysis, which can ultimately enhance the accuracy and comprehensibility of the outputs [65,66]. Predominant approaches in this regard are feature selection and feature extraction methods [67]. Given that AI/ML models are capable of identifying features in the data by associating the outcome and considering that we showed many metrics different than those traditionally known to precede cardiac arrest, feature selection techniques are deemed crucial to identify the most important variables as for model accuracy. In fact, as elucidated by Ladha et al. [68], feature selection in machine learning provides several benefits including the reduction of the dimensionality of the data space, and removing extraneous data, thereby improving data quality, optimizing storage requirements, and amplifying model efficiency and precision [68].

While integrating AI with telemetry data holds considerable potential, the presence of artifacts in telemetry data can hinder algorithm efficacy [69,70]. The development of AI models to integrate with telemetry findings largely relies on sources from clinical setting and public databases, utilizing primarily clinical and demographic data [71]. Leveraging these diverse datasets could enhance the detection of overlooked clinical scenarios, thereby improving the monitoring of vital signs through a more specific approach and establishing protocols for timely intervention. As an increasing number of datasets become available for AI algorithm modeling [72], the capacity of AI to predict CA will rapidly increase, allowing the leveraging of data specific to each clinical scenario, thereby increasing the accuracy and lead time for CA predictions.

### 4.10. Limitations of This Review

Our study had several limitations. First, the small number of studies included were all retrospective, limiting the ability to control bias and confounding variables. Indeed, we could not measure how the interventions before CA (e.g., vasoactive drugs and mechanical ventilation) modified the values of vital signs assessed. We excluded some studies that did not explicitly define the use of telemetry. In addition, IHCA is frequently studied as a part of a composite outcome, for which some studies did not fit our eligibility criteria. On the other hand, the methodology and objectives of each study varied significantly, deriving widely heterogeneous results that stopped us from conducting a metanalysis.

## 5. Conclusions

Our systematic review indicates that telemetry-detected vital signs hold the potential to predict IHCAs. We confirmed that the abnormalities of such digital biomarkers are highly frequent during hours before CA. However, we deem further research necessary to determine the single or combination of vital signs that most accurately predict IHCAs. Furthermore, the results of this review underscore the lack of consensus on how to best analyze the biomarkers detected by telemetry, emphasizing the need for standardized methodologies. Hence, this review stresses the need for more rigorous and larger-scale prospective studies to determine the predictive values of digital biomarkers and to support the development of accurate models. Provided that AI/ML-based-predicting models using vital signs and ECG metrics have been externally validated, the authors of this review contemplate them as promising, with an asset for supporting clinical decisions and improving outcomes, once externally validated.

## Figures and Tables

**Table 1:** Summary of included studies.

Author (Year)	Cases	Telemetry System	Physiological Markers Assessed	Time Assessed	Participant Characteristics	Controls	Types of CA
Do et al. (2015) [37]	81	General Electric monitoring systems (GE Healthcare, Waukesha, WI, USA),	ECG (5 metrics) •PR interval •QRS duration •ST segment elevation or depression •New onset arrhythmias •QTc (only for VT/VF/TdP arrests)	24 h	•Mean age (SD): 62 (19) •Male: 56% •ICU at the time of IHCA: 74%	No	•VT/VF/TdP: 18% •Bradyasystolic: 26% •PEA: 56%
Attin et al. (2015) [36]	39	Not reported	ECG (5 metrics) •Heart rate •QRS duration •ST segment elevation or depression •QRS morphology •New onset arrhythmias	8 h	•Mean age (SD): 69.5 (13) •Male: 64% •ICU at the time of IHCA: 44% •Structural heart conditions: 100%	No	•PEA and asystole: 100% (not specified) •VT/VF: excluded from the study
Churpek et al. (2012) [29]	88	Not reported	•Heart rate •Blood pressure (SBP and DBP) •Respiratory rate •Temperature •SatO_2_	48 h	•Mean age (SD): 64 (16) * •Female: 57% † •Prior ICU admission: 41% *	Yes	All ᵃ
Churpek et al. (2012) [30]	88	Not reported	•Heart rate •Blood pressure (SBP and DBP) •Respiratory rate •Temperature •SatO_2_	48 h	•Age, mean (SD): 64 (16) * •Female: 57% † •Prior ICU admission: 41% *	Yes, matched	All ᵃ
Hu et al. (2013) [32]	22	General Electric bedside monitors (GE Healthcare, Waukesha, WI, USA)	ECG (8 metrics) •Heart rate •PRp •pAmp •QTpc •tAmp •QRS width •rAmp •relJAmp	24 h	Mean age (SD): 63 (19) ‡ Male: 68% ‡ ICU at the time of CA: 77% ‡	Yes, matched	Bradysystolic CA
Rozen et al. (2014) [35]	36	Not reported	•Heart rate •Blood pressure (SBP and MAP) •Respiratory rate •SatO_2_	12 h	Age, median (IQR): 67 (52.5–75.0) † Male: 58.3% † ICU at the time of CA: 100% †	Yes, matched	•PEA: 38.9% •Severe bradycardia: 22% •Asystole: 11.1% •VT: 25% •Vfib: 2.8%
Ding et al. (2015) [31]	27	General Electric bedside monitors (GE Healthcare, Waukesha, WI, USA)	ECG (17 metrics) •PR interval •P-wave duration •QRS duration •RR interval •QT interval •SerumK2 •T-wave complexity •ST segment for leads I, II, and V •SDNN •HRV total power •HRV very low, low, and high •HRV normalized low and normalized high-frequency power	24 h	Mean age (SD): 61.4 (20.7) ‡ Male: 18 (66.7%) ‡ ICU at the time of CA: not reported ‡	Yes, matched	Bradyasystolic CA
Oh et al. (2016) [34]	46	GE Marquette Solar 8000M Patient Monitor (General Electric, Windsor, CT, USA)	•Heart rate •Blood pressure (SBP and DBP) •Respiratory rate •Temperature •SatO_2_	48 h	Mean age (SD): 61 (18) † Male: 72% † ICU at the time of CA: 100% †	Yes	All ᵃ ᵇ
McGrath et al. (2021) [33]	38	Masimo Root^®^ monitor (Masimo Corp., Irvine, CA, USA)	•Pulse rate •SatO_2_	12 h	Mean age (SD): 65.3 (12.9) † Male: 71.1% † ICU at the time of CA: 0%	Yes	PEA

* Significantly different from controls. † Not significantly different from controls. ‡ Significance not reported. ᵃ Shockable and non-shockable rhythms. ᵇ Only included patients who died of cardiac arrest. Abbreviations—CA: cardiac arrest, VT: ventricular tachycardia, VF: ventricular fibrillation, TdP: torsades de pointes, PEA: pulseless electrical activity, SBP: systolic blood pressure, DBP: diastolic blood pressure, MAP: mean arterial pressure, SD: standard deviation, IQR: interquartile range, PrP: interval from P peak to QRS onset, pAmp: peak amplitude of P wave, QTpc: interval from QRS onset to T peak corrected, tAmp: peak amplitude of T wave, rAmp: R-wave amplitude, relJAmp: relative absolute difference between ECG values at QRS onset and QRS offset, SerumK2: estimate of serum potassium using frontal leads, SDNN: standard deviation of normal to normal intervals, HRV: heart rate variability.

**Table 2:** Results of studies evaluating vital signs.

Study	SBP (mmHg)	DBP (mmHg)	MAP (mmHg)	Heart Rate (bpm)	Respiratory Rate (rpm)	Temperature (°C)	SatO_2_ (%)	Change from Baseline
Churpek et al. (2012) [29]	Not reported by authors †	* Minimum DBP achieved: Sen (95% CI) •<50: 45% (33–55) •<45: 29% (17–38) •<40: 16% (7–28) Spec (95% CI) •<50: 77% (57–84) •<45: 90% (79–96) •<40: 96% (92–99)	Not assessed	* Maximum HR achieved: Sen (95% CI) •>110: 44% (29–57) •>120: 28% (18–40) •>130: 19% (9–29) Spec (95% CI) •>110: 80% (70–88) •>120: 92% (81–98) •>130: 97% (92–99)	* Maximum RR achieved: Sen (95% CI) •>20: 67% (56–77) •>22: 49% (38–60) •>24: 32% (20–43) •>26: 25% (13–37) Spec (95% CI) •>20: 70% (51–80) •>22: 86% (78–92) •>24: 94% (89–97) •>26: 96% (92–99)	Not reported by authors †	Not reported by authors †	Change within case group from admission to 30 min prior to CA: •Heartrate: increased 9.6% (SD = 27) * •Respiratory rate: increased 9.3% (SD = 28) * Other vital signs not reported by authors †
Churpek et al. (2012) [30]	Not reported	Minimum achieved: CA group: •>49: 48% ‡ •40–49: 32% ‡ •35–39: 7% ‡ •<35: 14% ‡ Controls: •>49: 76% •40–49: 20% •35–39: 2% •<35: 2%	Not assessed	Maximum achieved: CA group: •<110: 47% ‡ •110–139: 36% ‡ •>139: 17% ‡ Controls: •<110: 76% •110–139: 22% •>139: 2%	Maximum achieved: CA group: •<21: 24% ‡ •21–23: 22% ‡ •24–25: 19% ‡ •26–29: 14% ‡ •>29: 22% ‡ Controls: •<21: 67% •21–23: 18% •24–25: 8% •26–29: 4% •>29: 2%	Not reported	Not reported	Not assessed
Rozen et al. (2014) [35]	Lowest achieved: median (IQR) •CA: 97 (80–106) * •Controls: 106 (90–115)	Not assessed	Lowest achieved: median (IQR) •CA: 65 (56–70) * •Controls: 70 (65–80)	Highest achieved: median (IQR) •CA: 110 (90–130) † •Controls: 100 (85–110)	Lowest achieved: median (IQR) •CA: 16 (12–20) * •Controls: 12 (10–16) Highest achieved: median (IQR) •CA: 22 (19–34) * •Controls: 18 (14–20)	Not assessed	Lowest oxygen saturation achieved: not reported by authors †	Not assessed
Oh et al. (2016) [34]	Mean value during 24 h before CA (SD) •Case group: 116.61 (15.08) ᵇ •Control 1 group: 104.85 (17.31) •Control 2 group: 132.82 (15.56) Mean value 1 h before the event (SD) •Case group: 75 (38.43) ‡ •Control 1 group: 60 (22.27) •Control 2 group: consistently higher	Mean value during 24 h before CA (SD) •Case group: 62.51 (10.99) ᵇ •Control 1 group: 58.02 (11.06) •Control 2 group: 77.17 (9.59) Mean value 1 h before the event (SD) •Case group: 43 (21.43) ‡ •Control 1 group: 35 (13.89) •Control 2 group: consistently higher	Not assessed	Mean value during 24 h before CA (SD) •Case group: 108.54 (19.98) ᵇ •Control 1 group: 103.63 (22.05) •Control 2 group: 84.33 (15.63) Mean value 1 h before the event (SD) •Case group: 83 (27.88) ‡ •Control 1 group: 62 (34.30) •Control 2 group: consistent, no prominent changes	Mean value during 24 h before CA (SD) •Case group: 24.64 (4.99) ᵇ •Control 1 group: 24.00 (5.36) •Control 2 group: 19.32 (3.76) Common pattern 1 h before CA: •Case group: wide fluctuations until shortly before cardiac arrest •Control 1 group: sudden drops from 2 h before death. •Control 2 group: consistent	Mean value during 24 h before CA (SD) •Case group: 36.25 (5.45) † •Control 1 group: 36.63 (0.62) •Control 2 group: 37.00 (0.39) Mean value 1 h before the event (SD) •Case group: 36 (5.40) ‡ •Control 1 group: maintained •Control 2 group: consistently higher	Not assessed	Not assessed
McGrath et al. (2021) [33]	Not assessed	Not assessed	Not assessed	(Pulse rate) Percent difference between the CA group and controls during the same interval: 0–5 min prior to CA: •Mean: 10.8% † •Range: 94.9% * 21–25 min prior to CA: •Mean: 7.6% † •Range: 20.9% †	Not assessed	Not assessed	Percent difference between the PEA group and controls during the same interval: 0–5 min prior to CA: •Mean: −7.2% * •Range: 185.9% * 21–25 min prior to CA: •Mean: −0.7% † •Range: 42.5% †	Percent difference within PEA group, 1 h vs. 7 h prior to CA Pulse rate: •Mean: −5.24% * •Range: −28.31% SatO_2_: •Mean: 2.7% * •Range: −33% *

* Significantly different from controls. † Not significantly different from controls. ‡ Significance not reported. ᵇ Significantly different from the control 2 group: non-CA subjects discharged after ICU. Abbreviations—SBP: systolic blood pressure, DBP: diastolic blood pressure, MAP: mean arterial pressure, CA: cardiac arrest, PEA: pulseless electrical activity, bpm: beats per minute, rpm: breaths per minute, SD: standard deviation, IQR: interquartile range, Sen: sensitivity, Spec: specificity.

**Table 3:** Results of studies evaluating ECG parameters.

Study	ECG Reading	Earliest Change	Heart Rate	Arrhythmias	PR Interval	QRS Duration	QRS Morphology	ST Segment	QT Interval	Other Parameters
Do et al. (2015) [37]	Manual	>1 h prior to CA for QRS prolongation, atrial tachy-arrhythmias, isorhythmic dissociation, and PR prolongation ≥50% when present	Not evaluated	Bradyarrhythmia: 28% of cases Non-sustained VT: 6% of cases Atrial tachyarrhythmia: 21% of cases (baseline Afib not included)	Shortening: 5% of cases Prolongation: 17% of cases	Prolongation: 19% of cases	Not evaluated	Elevation or depression: 31% of cases	QTc Prolongation: 50% of the VT/VF/TdP arrests Prolongation: 77% of the polymorphic VT/TdP arrests	N/A
Attin et al. (2015) [36]	Manual	Median time of QRS morphology changes: 121 min prior CA	•82% had decreased heart rate prior to CA. •The heart rate during the last hour before CA significantly differed from previous hours (*p* < 0.05). •Mean heart rate decreased by 3 bpm within the last hour (*p* < 0.01), 5 bpm within 10–5 min prior to CA (*p* < 0.01), and 7.6 bpm within the last 5 min (*p* < 0.01).	Observed during periods of decreased heart rate: •Sinus bradycardia: 47% •Junctional rhythm: 28% •Afib: 19% •3rd-degree AV block: 6%	Not evaluated	Prolongation in 51% of cases. More often (*p* < 0.01) in the last hour vs. 8 h before CA.	QRS morphology changes: •Overall: 54% of cases •Asystole arrests: 80% •PEA arrests: 38%	Elevation: 51% of cases Depression: 59% of cases	Not evaluated	N/A
Hu et al. (2013) [32]	Automated	Longest lead time was 20.4 h (SD = 1.6) for QRS width ≥184.8 ms or ≤73.8 ms with a TPR = 9.1% and FPR = 0%	Metric value: •≥149.3 bpm: TPR = 4.6% FPR = 0% •≤38.8 bpm: TPR = 4.6% FPR = 0% Absolute slope of the trending (1-h window) •TPR = 9.1% FPR = 0%	Not evaluated	PRp Metric value: •≥196.6 ms: TPR = 27.3% FPR = 0% Absolute slope of the trending (0.5-h window) •TPR = 13.6% FPR = 0%	Metric value: •≥184.8 ms: TPR = 13.6% FPR = 0% •≤73.8 ms: TPR = 9.1% FPR = 0% Absolute slope of the trending (2-h window) •TPR = 13.6% FPR = 2.3%	Not evaluated	Not evaluated	QTpc Metric value: •≥533.8 ms: TPR = 9.1% FPR = 0% •≤229.9 ms: TPR = 9.1% FPR = 2.3% Absolute slope of the trending (1-h window) • TPR = 13.6% FPR = 2.3%	Metric value: •relJAmp ≥ 20%: TPR = 22.7% FPR = 0% •tAmp ≥ 3.9 au: TPR = 9.1% FPR = 0% •rAmp ≥ 9.3 au: TPR = 4.6% FPR = 2.2% •rAmp ≤ 0.9 au: TPR = 4.6% FPR = 0% •pAmp ≥ 2.7 au: TPR = 4.6% FPR = 2.3% •pAmp ≤ 0.1 au: TPR = 4.6% FPR = 0% Absolute slope of the trending (2-h window): •relJAmp: TPR = 18.2% FPR = 2.3% •tAmp: TPR = 4.6% FPR = 0% •rAmp: TPR = 27.3% FPR = 2.3%
Ding et al. (2015) [31]	Automated	Longest lead time was 16.1 h (SD = 8.1) for ST II ≥ 143 μV with a TPR = 7.4% ᵃ	Not evaluated	Not evaluated	Trend duration: •2.8 h: TPR = 48.2% ᵃ Slope: •−8.8 ms/h: TPR = 18.5% ᵃ •17.5 ms/h: TPR = 18.5% ᵃ	Trend duration: •2.7 h: TPR = 40.7% ᵃ Slope: •7.4 ms/h: TPR = 14.8% ᵃ	Not evaluated	Trend duration: •ST I (3.0 h): TPR = 51.9% ᵃ •ST II (3.3 h): TPR = 18.5% ᵃ •ST V (3.1 h): TPR = 33.3% ᵃ Terminal value: •ST I ≤ −56 μV: TPR = 22.2% ᵃ Slope: •ST I = 8.8 ms/h: TPR= 14.8% ᵃ •ST II = −14.6 ms/h: TPR = 14.8% ᵃ •ST II = 37.6 ms/h: TPR = 14.8% ᵃ •ST V = 32.6 ms/h: TPR = 14.8% ᵃ	Trend duration: •3.3 h: TPR = 33.3% ᵃ Terminal value: •≤313 ms: TPR = 11.1% ᵃ •≥492 ms: TPR = 11.1% ᵃ Slope: •−23.5 ms/h: TPR = 11.1% ᵃ •19.4 ms/h: TPR = 25.9% ᵃ	Trend duration: •RR (3.5 h): TPR = 51.9% ᵃ •T Complex (2.9 h): TPR = 40.7% ᵃ •SDNN (3.6 h): TPR = 40.7% ᵃ •SerumK2 (3.2 h): TPR = 29.6% ᵃ •Pdur (2.7 h): TPR = 25.9% ᵃ •HRV (3.0 h): TPR = 25.9% ᵃ

ᵃ At FPR = 5%. Abbreviations—CA: cardiac arrest, VT: ventricular tachycardia, VF: ventricular fibrillation, TdP: torsades de pointes, PEA: pulseless electrical activity, Afib: atrial fibrillation, bpm: beats per minute, SD: standard deviation, TPR: true positive rate, FPR: false positive rate, PrP: interval from P peak to QRS onset, pAmp: peak amplitude of P wave, QTpc: interval from QRS onset to T peak corrected, tAmp: peak amplitude of T wave, rAmp: R-wave amplitude, relJAmp: relative absolute difference between ECG values at QRS onset and QRS offset, Pdur: P-wave duration, RR: interval from R peak to R peak, SerumK2: estimate of blood potassium using frontal leads, T Complex: T-wave complexity, ST I: ST segment levels for leads I, ST II: ST segment levels for leads II, ST V: ST segment levels for leads V, SDNN: standard deviation of normal to normal intervals, HRV: heart rate variability total power (<0.4 Hz), ms: milliseconds, ms/h: milliseconds per hour.

## Data Availability

The databases generated for drafting this manuscript can be solicited from the author upon reasonable request.

## Protocol Registration

This systematic review was not registered due to the rapidly evolving nature of research in predicting cardiac arrest, necessitating immediate and flexible research approaches. Rigorous methodological standards were maintained to ensure the integrity of the review.

## Supplementary Materials

The following supporting information can be downloaded at: https://www.mdpi.com/article/10.3390/jcm12237430/s1, File S1: Search strategy; PRISMA_2020_checklist_GH_20211209. Reference [73] is cited in the Supplementary Materials.
