# Supplementary material for: Predicting Cardiopulmonary Arrest with Digital Biomarkers: A Systematic Review
Source: J Clin Med. 2023 Nov 30;12(23):7430. doi: 10.3390/jcm12237430 (PMC10707115; doi:10.3390/jcm12237430)
Supplement: Supplementary file 1 [file jcm-12-07430-s001.zip › Supplementary File S1 - Search strategy.pdf]

Supplementary File S1

PubMed: search performed on 11/07/2022 yielding 192 results

> (("Monitoring, Physiologic"[Mesh:NoExp] OR "telemetry"[Mesh:NoExp] OR "Hemodynamic Monitoring"[Mesh] OR "continuous monitoring"[tw] OR "oximetr*"[Mesh] OR "Vital Signs"[Mesh] OR "electrocardiog*"[tw]) AND ("Early Diagnosis"[Mesh:NoExp] OR "Diagnosis, Computer-Assisted"[Mesh:NoExp] OR "Diagnosis"[Mesh:NoExp] OR "detect*"[tw] OR "identification"[tw])) AND ("Death, Sudden, Cardiac"[Mesh:NoExp] OR "sudden cardiac arrest"[tw] OR "Heart Arrest"[Mesh] OR "Cardiopulmonary arrest"[tw] OR "cardiac arrest"[tw] OR "in-hospital cardiopulmonary arrest"[tw] OR "impending cardiac arrest"[tw]) AND ("Inpatients"[Mesh] OR "Inpatient*"[tw] OR "in-hospital"[tw] OR "Emergency department"[tw] OR "ICU"[tw] OR "intensive care unit")

Google Schoolar: search performed on 11/10/2022 yielding 1080 results. (10 first pages were screened)

> "vital signs" AND "continuous monitoring" AND "Cardiac arrest" AND inpatients AND prediction -remote -ambulatory

EMBASE: search performed on 11/14/2022 yielding 130 results.

1) clinical monitoring/ or apnea monitoring/ or alarm monitoring/ or body temperature monitoring/ or hemodynamic monitoring/ or monitoring/ or blood pressure monitoring/ or physiologic monitoring/ or cardiac stress monitoring system/ or patient monitoring/ or vital sign/ or telemetry/ or pulse oximetry/ or oximetry/ or heart rate/ or electrocardiogram/ 755843
2) diagnosis/ or early diagnosis/ or prediction/ or detect*.mp. 5219058
3) exp hospital patient/ or emergency patient/ or hospital emergency service/ or ward/ 253519
4) sudden cardiac death/ or heart arrest/ or cardiopulmonary arrest/ or sudden arrhythmic death syndrome/ 106065
5) 1 and 2 and 3 and 4 130

Web of sciences: search performed on 11/16/2022 yielding 223 results.

> (((ALL=("Monitoring, Physiologic" OR "telemetry" OR "Hemodynamic Monitoring" OR "continuous monitoring" OR "oximetr*" OR "Vital Signs" OR "electrocardiog*")) AND ALL=("Early Diagnosis" OR "Diagnosis, Computer-Assisted" OR "Diagnosis" OR "detect*" OR "identification")) AND ALL=(("Death, Sudden, Cardiac" OR "sudden cardiac arrest" OR "Heart Arrest" OR "Cardiopulmonary arrest" OR "cardiac arrest" OR "in-hospital cardiopulmonary arrest" OR "impending cardiac arrest")) AND ALL=("Inpatient*" OR "in-hospital" OR "Emergency department" OR "ICU" OR "intensive care unit"))
